# Psychological and Physiological Processes in Figure-Tracing Abilities Measured Using a Tablet Computer: A Study with 7 and 9 Years Old Children

**DOI:** 10.3389/fpsyg.2016.01528

**Published:** 2016-10-18

**Authors:** Enrico Giammarco, Sergio Di Sano, Tiziana Aureli, Paola Cerratti, Giorgio Fanò-Illic, Tiziana Pietrangelo

**Affiliations:** ^1^Department of Neuroscience, Imaging and Clinical Sciences, University ‘G. d’Annunzio’ of Chieti-PescaraChieti, Italy; ^2^Laboratory of Functional Evaluation, University ‘G. d’Annunzio’ of Chieti-PescaraChieti, Italy

**Keywords:** tablet computer, figure tracing, handwriting, visual–motor skills, visual–spatial skills

## Abstract

The present study investigated the use of a tablet computer to assess figure-tracing skills and their relationships with psychological (visual–perceptual processes, cognitive processes, handwriting skills) and physiological (body mass index, isometric strength of arms) parameters with school-children of second (7–8-year-olds) and fourth (9–10-year-olds) grades. We were also interested in gender differences. The task required tracing of geometric figures on a template, shown on a tablet screen in light gray, for the segments that make up the target figure, one at a time. This figure-tracing tablet test allows acquisition and automated analysis of four parameters: number of strokes (pen lift) for each segment; oscillations of lines drawn with respect to reference lines; pressure of pen on tablet; and average speed of tracing. The results show a trade-off between speed and quality for the tablet parameters, with higher speed associated with more oscillations with respect to the reference lines, and lower number of strokes for each segment, in both male and female children. The involvement of visual–motor integration on the ability to reduce the oscillations in this tablet test was only seen for the male children, while both the male and female children showed a relationship between oscillations and more general/abstract visual–spatial processes. These data confirm the role of visual–motor processes in this figure-tracing tablet test only for male children, while more general visual–spatial processes influence the performance in the tablet test for both sexes. We conclude that the test proposed is useful to screen for grapho-motor difficulties.

## Introduction

In the western literate community, handwriting is a one of the most important skills that children acquire during their primary-school years. Despite the increasing popularity of digital tools that are based on typing keys and touch screens, handwriting still has great importance in the school context. Indeed, children spend a lot of time in activities that require writing, and this ability is associated with success in school, while difficulties in writing are associated with lower self-esteem (Feder and Majnemer, 2007).

Writing involves integration of perceptual and cognitive abilities that develop during the preschool period, and that consolidate from 7 to 9 years of age, during primary school. In the preschool years, the sensory-motor integration involved in copying letters is the most important process (Daly et al., 2003). In primary school, children develop the ability to automate handwriting through construction of mental images of letters and learning of the movements involved in writing (Chartrel and Vinter, 2008; Scordella et al., 2015).

Writing involves both high-level and low-level processes. High-level processes are at work in the composition of the writing, while low-level processes are involved in the writing process itself (Berninger et al., 1997). These different processes require the following: fine motor control; bilateral and visual–motor integration; motor planning; in-hand manipulation; proprioception; visual perception; sustained attention; and sensory awareness of the fingers (Feder and Majnemer, 2007). Indeed, writing requires a high level of coordination and high precision-force regulation. Thus, strength is one of the most important physiological parameters under investigation in handwriting ability.

Difficulties in writing are typically associated with problems with fine motor skills, which appear to be due to increased levels of neuromotor noise that are compensated for by increased phasic stiffness of the limb system (Smits-Engelsman et al., 2001). Writing deficits can also originate, at least in part, from a low-level deficit in the primary process of rapid processing of visual stimuli, which involves visual attention (Franceschini et al., 2012) and/or magnocellular–dorsal activation (Gori et al., 2014), as has been reported for dyslexia (Gori and Facoetti, 2015; Gori et al., 2015). A low-level deficit in this process can produce cascade effects on writing abilities.

Handwriting skills are evaluated using two methods that are based on the analysis of writing production and speed, or of the handwriting process (Rosenblum et al., 2003b). Assessing writing as a product involves analytic evaluation of its readability, and the most used tests for this are the *Children Handwriting Evaluation Scale* (CHES), the *Concise Evaluation Scale for Children’s Handwriting* [*Beknopte beoordelingsmethode voor kinderhandschriften* (BHK); also available for written Italian; Di Brina and Rossini, 2011], and the *Hebrew Handwriting Evaluation* (HHE; for written Hebrew). For Italian people, there is also the *Test for Evaluation of Grapho-Motor and Postural Difficulties in Writing* [*Test per la Valutazione delle Difficoltà Grafo-Motorie e Posturali della Scrittura* (DGM-P); Borean et al., 2012]. These scales assess writing readability through qualitative criteria, in terms of the size, slant, spacing, shape, and line straightness (Rosenblum et al., 2003b), and they provide data that vary according to the nature of the task and the type of instructions. In addition, they do not always specify who is qualified to administer these scales (e.g., teacher, therapist, others). Finally, the coding of the responses is also costly in terms of time, and is not always precisely defined.

These scales refer to the written output (the product) and not to the process of handwriting. However, functional handwriting should not only be legible, but should also be performed in a reasonable amount of time. For this reason, methods based on the writing process measure such specific variables while the child is actually performing a writing task, such as time, space, and pressure (Rosenblum et al., 2003b). These methods involve the assessment of writing speed, which is analyzed in different ways, and the results do not always overlap across the different methods.

Analysis of the writing process can be carried out using a digitizing graphics tablet that is provided with a special pen and a computer that records the ‘x’ and ‘y’ coordinates of the pen on the graphics tablet. Such recordings can allow information to be obtained for the spatial and temporal features of handwriting in real time. This procedure was developed between the 1980 and 1990s, and at first it was mainly used for adults, gradually extending only later to children (Van Gemmert and Teulings, 2006). The use of a graphics tablet allows a number of variables to be taken into consideration, some of which are different from those obtained using a paper and pencil test. These can be assessed with greater accuracy and objectivity, and they include the following: variability, accuracy, speed, pressure, and number of components/strokes.

The first studies on a computer-monitored graphics tablet with writing tasks started about 20 years ago. Van Galen et al. (1993) studied school-children from second to fourth grade (7–10 year olds) with a writing task that involved writing meaningless words. They reported that the less proficient children produced larger movements with greater speed. Their conclusion was that poor writers are less effective in inhibition of neuromotor noise. Smits-Engelsman and van Galen (1997) then used a longitudinal design with primary-school children (6–11 year olds) who were tested with writing tasks that were recorded on a computer-monitored graphics tablet. The task involved the production of letter strings of varying complexities. The movements of the poor writers were substantially more ‘noisy’ than those of the proficient writers, with a noise peak in the region of neuromotor tremor. In addition, their movements were also less accurate. The conclusion was that poor writers lack control of spatial accuracy.

Other authors collected digitizer-based measures from children in primary school using graphics tablets, and they highlighted some characteristics of the poor writers with respect to the proficient writers. In particular, Smits-Engelsman et al. (2001) concluded that the “poor writers choose to apply higher phasic stiffness to filter higher neuromotor noise in the neuromotor apparatus” (Smits-Engelsman et al., 2001). Rosenblum et al. (2003a) concluded that non-proficient handwriters required significantly more time to perform the writing tasks. Di Brina et al. (2008) showed higher variability in the letter forms of poor writers compared to good writers. Accardo et al. (2013) implemented an algorithm to analyze the parameters of length, duration, and speed of a written trace for a sentence transcription task, and they reported an improvement in motor automation with age (i.e., reduction in the number of strokes per letter). Recently, Pagliarini et al. (2015) explored the hypothesis that the relationship between poor handwriting and dyslexia is mediated by rhythm. Falk et al. (2011) described a portable computer-based handwriting assessment tool to objectively measure Minnesota Handwriting Quality scores and to detect writing difficulties in children. Kushi et al. (2011) studied biomechanics changes to handwriting over extended writing periods. Alamargot and Morin (2015) compared grapho-motor execution of handwriting using a plastic-tipped pen on the screen of a digital tablet with a ballpoint pen on paper.

Some studies have used very simple graphic tasks that do not involve the writing of single letters or an extended text, but simple graphics patterns. These studies have provided important information for the development of both writing and drawing skills. Lord and Hulme (1988) reported that drawing performance is related to visual discrimination in clumsy children, but not in a control group of typical children. Lange-Küttner et al. (2002) reported an increase in the ability for children to draw a realistic contour during human figure drawing when in their primary-school years.

Differences in handwriting between male and female children have been shown in some more recent studies (Hystegge et al., 2012; Lange-Küttner and Ebersbach, 2012; Tabatabaey-Mashadi et al., 2015). Tabatabaey-Mashadi et al. (2015, p. 25) analyzed strategies of digitizing tablet drawing by 6–7-year-old children, and they reported that when these children draw a rhomboid, compared to boys, “girls pay more attention to details, and they tend to perform the polygon segment by segment rather than drawing it with fewer strokes.” Lange-Küttner and Ebersbach (2012) used a drawing cube task, and they reported that female children focus more on detail while male children focus more on shape. In a study on reading skills of 10-year-old children, Hystegge et al. (2012, p. 123) reported that “boys and girls differ with respect to the correlation between visual processing skills and reading performance” which confirmed the hypothesis based on the male advantage for visual memory skills.

Little is known about the role of muscular strength and arm pressure on writing skills and drawing. Toomela (2002) investigated several factors that can influence the drawing development in 2-year-old to 11-year-old children, including motor abilities. In a copy task of a basic graphic pattern, Lange-Küttner (1998) showed an increase in pressure between 4 and 6 year olds as a result of greater muscular tension when drawing.

Our study adds to those that have used tablets, by measuring the figure-tracing skills in a group of 7–10-year-old children. This differs from previous studies, as due to recent technological advances that have enabled tablet computer to reach performance standards that are comparable to graphics tablets, a tablet computer was used instead of a digitizing tablet connected to a computer. This choice was made possible by the use of a tablet with an electronic pen that allowed simulation of writing with paper and pen, by writing directly on the screen of the device. Moreover, through the implementation of dedicated software, this tablet automatically analyzed the process of figure tracing and extracted the parameters of the graphic performance. Therefore, this tablet computer is easier to use in practice than a graphics tablet.

Another difference was the use of a simple tracing task. This task required the drawing of simple geometric figures on top of a template (i.e., the reference figures, shown on the tablet in light gray), by tracing the segments (as eight lines) that made up the target figure, one at a time. The stimuli for each trial were two shapes (i.e., square, diamond) composed of segments with different orientations (i.e., vertical, horizontal, oblique). The task involved tracing over these segments in both directions (from left to right, and right to left). This task can be used to assess elementary processes involved in grapho-motor aspects of handwriting (and drawing).

Four parameters were used that were obtained in these figure-tracing tablet computer tests: number of components for each segment (strokes, defined by the pen lift); oscillations of the line drawn with respect to the reference line; pressure of the pen on the tablet (without taking into account the orientation of the hand); and mean speed of writing (weighted, taking into account the length of the components of the segments tracked).

We were mainly interested in studying the relationships between the indices provided by the tablet and those obtained from a paper and pencil test (i.e., the DGM-P), relative to speed and readability. We were also interested in investigating the relationship between the tablet indices and the other processes involved in writing; i.e., visual–motor integration, visual–spatial skills, and other cognitive skills (e.g., memory, planning). Finally, we measured the physiological parameters of maximal voluntary isometric contraction of the child’s arm, to correlate this with the pressure developed during this figure-tracing tablet test.

The present study investigated four main hypotheses. The first of these involved the relationships between the four indices of the tablet test: i.e., number of strokes, oscillation with respect to the reference line, pressure, and speed, in particular as a trade-off between accuracy (oscillations) and speed. We expected an age effect for the oscillation parameter, with fewer oscillations in older children. We also expected an effect of sex, with fewer oscillations for the male children due to their higher visual–spatial abilities with respect to the female children.

The second hypothesis involved the relationships between physiological strength of the arm and the pressure exerted by the pen on the tablet. We expected greater isometric force in the arm to be associated with greater pressure of the pen. We also expected a sex effect here, as a stronger relationship for male children than for female children, due to their greater muscle mass.

The third hypothesis involved the relationships between the indices of the figure-tracing tablet test and the indices of the paper and pencil handwriting tests (i.e., sentence copying test, praxis test). We expected that the indices of accuracy/legibility and spatial-metric variation for the sentence copying test would correlate with accuracy (oscillations of the line tracing) for the figure-tracing test on this tablet. We also expected a relationship between the speed parameter in the tablet test and the velocity in the sentence-copying task, because both of these involve the ‘best condition’ (i.e., with instructions for the children to do their best, with no hurry). Finally for these comparisons, we expected a relationship between the tablet parameters and the handwriting fluency (praxis) test, although this would be weak, as the praxis test involves the ability to produce recognizable graphemes in a short time (i.e., without the formal precision of letters).

The fourth hypothesis involved the relationship between the figure-tracing tablet test and other processes, in terms of visual–motor integration, and visual–spatial processes and planning. We expected the visual–motor/visual–spatial processes to be involved in the figure-tracing skill only for male children (see Hystegge et al., 2012), while more general cognitive processes should be involved in the figure-tracing tests for both sexes (Scordella et al., 2015).

## Materials and Methods

### Participants

The participants were recruited from three different schools, after permission was granted by the school administration and the parents of the children. Children with a certificate of disability were excluded from the sample. The 83 children assessed were between 7 and 10 years of age (mean age, 8.5 ± 1.1 years), and comprised 41 males (mean age, 8.5 ± 1.0 years) and 42 females (mean age, 8.5 ± 1.1 years). Of the males, we analyzed 24 in the second grade (mean age, 7.6 ± 0.36 years), and 17 in the fourth grade (9.5 ± 0.30 years). Similarly, for the females, there were 25 in the second grade (7.5 ± 0.38 years) and 17 in the fourth grade (9.4 ± 0.30 years). The children were tested at their school by the psychologists and physiologists, and in the presence of the teachers, who participated in the project. The tests were carried out on different days/sessions over a few weeks.

The children were measured in terms of their anthropometric characteristics, including weight, height, BMI (weight divided by height squared [kg/m^2^]), and maximum voluntary contraction of the arm (kg) (**Table 1**).

**Table 1:** The physiological parameters of the children in the second (II) and fourth (IV) grades of the primary schools who participated in this study.

Primary school grade (expected age range)	Gender (*n*)	Age (years)	Body mass index (kg/m^2^)	Maximum voluntary contraction (arm; kg)
II (7–8 years)	Male (24)	7.6 ± 0.4	18.7 ± 3.5	15.2 ± 2.6
	Female (25)	7.5 ± 0.4	16.9 ± 2.2	14.2 ± 1.7
IV (9–10 years)	Male (17)	9.5 ± 0.3	20.4 ± 3.3	17.8 ± 5.3
	Female (17)	9.5 ± 0.3	19.6 ± 3.3	20.0 ± 3.8

*Data are mean ± standard deviation*.

The study was approved by the Ethics Committee of the ‘G. D’Annunzio’ University of Chieti-Pescara, and informed consent was signed by the parents of the children. The study conformed to the Declaration of Helsinki.

### Assessment of the Figure-Tracing Tablet-Computer Test

**FIGURE 1:**
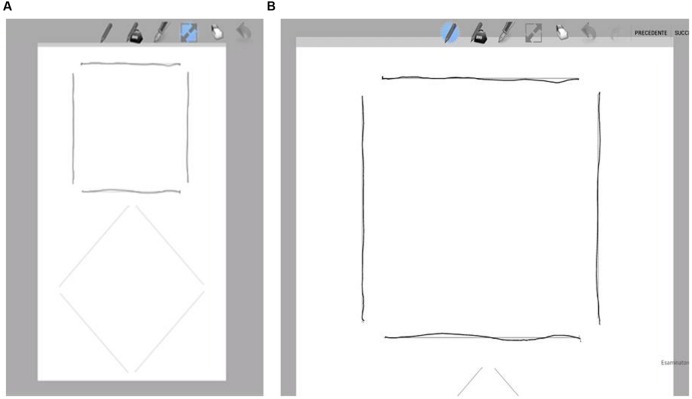
**The tablet-computer screen for the tablet test. (A)** The blank screen on which a square and a diamond were represented in light gray, as shown for the tablet screen. **(B)** During the test, the sheet in the screen was zoomed in so that the square or the diamond were large enough to cover the whole of the screen. The children were required to trace the segments by drawing along the gray lines on the screen, as shown.

A blank sheet was shown on the tablet screen on which a square and a diamond were represented in light gray, in four segments that were not connected at the top (**Figure 1A**). The children were required to redraw the segments by drawing along the gray lines on the screen. During the test, the sheet in the screen was zoomed in, so the square and the diamond were large enough to cover the entire screen. **Figure 1B** shows an example of how the screen appeared during the tests.

The children performed the tablet test sitting at a school desk next to the examiner. The test was preceded by a familiarization phase, during which the children practiced drawing lines with the pen on the tablet. Then the test began, during which the tablet could not be moved from the original position (i.e., parallel to the edge of the desk). The test consisted of tracing the segments (from left to right) one at a time, always starting from the same segment and proceeding clockwise. The test was repeated four times, with a short pause between the repetitions. The task required the tracing of the segments without any hurry and as well as possible (i.e., under best conditions).

We calculated the mean over the second and third repetitions for the analysis. We excluded the first repetition as familiarization. In a study by Lange-Küttner et al. (2014), the repetition of drawings in a Draw-A-Person (DAP) test produced an increase in the omission of detail, but the introduction of a novel task recovered the scores. So the risk of having more repetitions was to make the task boring or less interesting. However, this effect was not seen in the present study. Based on an analysis of ANOVA for repeated measures, the fourth repetition gave results that were similar to the second and third repetitions, although it lengthened the time of administration of the test, so it was excluded from the analyses, to prevent any fatigue effects for the children. In the future, we would recommend only three repetitions of such a task.

The tablet test was assessed according to the following four indices.

#### Number of Strokes

All lines drawn with the stylus on the tablet screen were stored individually as software objects, or ‘traits,’ that we refer to here as ‘strokes.’ As the tip of the pen touched the display until it was lifted, the coordinates of the contact points of the stylus on the screen were acquired from the touch-screen and stored by the software.

#### Oscillation of the Graphic Line

For each stroke, the geometric coordinates of the stroke points were stored and the quadratic deviation of the points from the ideal lines (i.e., the gray lines) were calculated, in millimeters.

#### Pressure

For each stroke, the pressure exerted by the pen was calculated as a weighted average over the length. In particular, the touch-screen of the tablet provided a measure of the pressure of the pen on the surface of the display, calculated as the deflection of the layers according to the pressure exerted. The pressure was then made available by the software as a whole number that varied between 0 and 1023. The software then stored this number along with the coordinates of each sampled point.

#### Speed

For each stroke, the time of the start and end of the tracking were acquired. For each stroke, the speed was calculated as the weighted average over the length, in mm s^-1^.

### Assessment of Handwriting

Two tests were used to assess the quality and efficiency of the handwriting: the Praxis subtest of the test for the evaluation of orthographic competence and writing [*Batteria per la Valutazione della Scrittura e della Competenza Ortografica* (BVSCO-2)] and the DGM-P, as defined below.

#### Praxis of Writing

The praxis of writing was examined according to the BVSCO-2 manual (Tressoldi et al., 2013), using three writing tasks: (a) writing the sequence of letters “*le*” (as lower-case cursive characters) for 1 min (i.e., the LE praxis); (b) writing the sequence of letters “*uno*” (i.e., one; as lower-case cursive characters) for 1 min (i.e., the UNO praxis); and (c) writing the sequence of numbers “*uno*-*due*-” (i.e., one-two-) and so on for 1 min (i.e., the number praxis). The test involved calculation of the measure of fluency, as how many graphemes were written correctly in 1 min.

#### Grapho-Motor Difficulties in Writing

The DGM-P test is an assessment tool for grapho-motor and postural difficulties in writing (Borean et al., 2012). The DGM allows the assessment of cursive handwriting in children from second to fifth grade in primary schools (7–11 year olds). The test requires two transcripts to be performed in succession in cursive, of a simple sentence written in lowercase letters, and according to two different conditions: one focused on the accuracy of the writing (i.e., ‘best writing’), and the other focused on the speed of the writing (i.e., ‘fast writing’). The analyses reported here are based only on the ‘best writing’ condition, to have data comparable with those provided by the figure-tracing tablet test.

The DGM-P can quantify 12 variables (i.e., indices, parameters) that characterize the efficiency (or inefficiency) of writing, including speed of execution of the task, and readability of the written text. Here, we analyzed 11 of these performance indices under the ‘best writing’ condition, as: (i) ‘Speed’; (ii) ‘Self-correction’; (iii) ‘Fluctuating letters’; (iv) ‘Dysmetria’; (v) confusion between similar letters (‘Confusion letter’); (vi) ‘Size of ascending and descending segments of letters’; (vii) ‘Unrecognizable letters’; (viii) ‘Insufficient space between letters’; (ix) maximum amplitude of fluctuation between letters (‘Amplitude fluctuation’); (x) ‘Maximum variation in height of mean letters’; and (xi) ‘Maximum variation in height of ascending and descending segments of letters’. We excluded the parameter ‘Learning errors.’

These 11 measures were grouped according to three categories: (a) ‘Speed index’ (Speed); (b) ‘Spatial-metric variation index’ (Amplitude fluctuation, Maximum variation in the height of mean letters, Maximum variation in the height of ascending and descending segments of letters); and (c) ‘Legibility of letters index’ (Self-correction, Fluctuating letters, Dysmetria, Confusion letter, Size of ascending and descending segments of letters, Unrecognizable letters, Insufficient space between letters). The Speed Index is given by the number of letters written per second, the spatial-metric variation index is given in mm, and the Legibility of letters index involved counting the number of incorrect letters for each parameter.

### Assessment of Visual–Motor Integration

The visual–motor integration test is a paper and pencil test in which children have to copy geometric forms that become progressively more difficult (Beery and Buktenica, 2000). The aim was to measure the visual–motor integration in terms of the ability to control hand movements guided by vision. These data were analyzed using standard scores.

### Assessment of Cognitive Abilities

The *Kaufman Assessment Battery for Children*, second edition (KABC-II) (Kaufman and Kaufman, 2010) is a battery of tests that measures the cognitive abilities and processes of children, based on two theoretical models of how the mind works: the psychometric model of specific cognitive abilities of Cattel–Horn–Carroll (Kaufman and Kaufman, 2010); and the neuropsychological model of Luria (Kaufman and Kaufman, 2010). The complete battery includes 18 tasks, but we administered the six tasks that were more related to the abilities we considered as key for the motor-coordination assessment (see Introduction). These were grouped according to three subtests, in terms of sequential (memory), planning (reasoning), and simultaneous (visual–spatial) processes, according to the ages of the children, as a scaled score.

The sequential processes included: (i) ‘Number recall’: the children had to repeat a series of numbers in the same order in which the psychologist said them; and (ii) ‘Word order’: the children had to indicate the figures in a series of drawings that represented objects according to the instructions of the examiner, and respecting the order.

The planning processes included: (i) ‘Pattern reasoning’: the children had to complete the set of a series of stimuli that formed a logical sequence by choosing the correct item among those proposed; (ii) ‘Story completion’: the children had to choose the picture that completed a story that was presented by the pictures, where some of the pictures were missing.

The simultaneous processes included: (i) ‘Rover’: the children had to move a toy dog on a piece of cardboard divided into small squares while looking for the fastest route to reach the destination and using the lowest number of movements; (ii) ‘Triangles’: the children had to assemble some triangles to form a figure.

These data were analyzed using standard scores, both for the three processes and for the six tasks. We were especially interested in the Triangles task, as a measure of visual–spatial skill.

### Statistical Analysis

Statistical analysis was performed using IBM SPSS Statistics, version 20. Statistical comparisons were calculated using Spearman correlations, and 95% confidence limits. Two-tailed ANOVA with repeated measures was used to analyze the repeatability of the measures for the four repetitions of the tablet test, and for the descriptive statistics of the tablet parameters. The data are presented as mean ± standard deviation (SD). Significance was based on *p* < 0.05, with significant values in the data presentation highlighted in bold.

## Results

### Analysis of the Figure-Tracing Tablet-Computer Test

To analyze the effects of age and sex on the four parameters of the tablet, we performed four analyses of variance 2 (AGE) × 2 (SEX). The ANOVA for the oscillation parameter showed a significant main effect of AGE [*F*(1,79) = 7.31, *p* = 0.008], which was modulated by the interaction AGE × SEX [*F*(1,79) = 4.15, *p* = 0.045]. As shown in **Table 2**, the oscillations decreased with age for the male children, but not for the female children.

**Table 2:** The computer tablet parameters of the male and female children in the second (II) and fourth (IV) grades of the primary schools.

Gender	Grade (*n*)	Strokes	Oscillations	Pressure	Speed
Male	II (24)	17.8 ± 8.5	0.55 ± 0.13	60 ± 11	14.9 ± 4.6
	IV (17)	20.4 ± 7.4	0.44 ± 0.09	65 ± 9	12.9 ± 4.2
	II+IV (41)	18.9 ± 8.1	0.51 ± 0.13	62 ± 10	14.0 ± 4.5
Female	II (25)	16.5 ± 4.7	0.47 ± 0.10	64 ± 10	13.4 ± 3.8
	IV (17)	16.2 ± 3.7	0.46 ± 0.07	68 ± 8	13.9 ± 3.9
	II+IV (42)	16.4 ± 4.2	0.46 ± 0.09	66 ± 9	13.6 ± 3.8

*Data are mean ± standard deviation*.

The ANOVA for the stroke parameter showed no significant main effects, nor interaction effects. The ANOVA for the pressure parameter showed a marginally significant effect of AGE [*F*(1,79) = 4.09, *p* = 0.047], with greater pressure for the older children. The main effect of SEX and the effects of the interaction AGE × SEX were not significant. Finally, no significant effects were seen for the ANOVA for the speed parameter.

To analyze the relationships between the tablet parameters, we carried out separate correlational analysis for the male and female children (**Table 3**). The results showed that the oscillations and strokes were negatively correlated, as well as the speed and strokes, while the speed and oscillations were positively correlated. The data were similar for the male and female children.

**Table 3:** Correlations between the four tablet-computer parameters.

	Strokes		Oscillations		Pressure	
	ρ	*p*	ρ	*p*	ρ	*p*
**Oscillations**						
Male	**-0.55**	*0.0001*				
Female	**-0.65**	*0.0001*				
**Pressure**						
Male	-0.17	*0.27*	-0.06	*0.7*		
Female	-0.07	*0.66*	-0.11	*0.47*		
**Speed**						
Male	**-0.82**	*0.0001*	**0.71**	*0.0001*	-0.02	*0.88*
Female	**-0.81**	*0.0001*	**0.73**	*0.0001*	-0.06	*0.7*

*Significant values (p < 0.05) highlighted in bold. p values reported in italic.*

We examined in detail the role of the age in handwriting development, where the oscillations and stroke parameters were negatively correlated for both the second-grade and fourth-grade children (*r* = -0.58, *r* = -0.59, respectively; *p* = 0.001 for both). The speed parameter of the tablet test was correlated with oscillations for the children of both grades (*r* = 0.74, *p* = 0.001), and with strokes for the children of the second and fourth grades (*r* = -0.84, *r* = -0.74, respectively; *p* = 0.001 for both). The pressure parameter of the tablet test was correlated with strokes only for the children of the fourth grade (*r* = -0.35, *p* = 0.04).

### Assessment of Physiological Parameters and the Figure-Tracing Tablet-Computer Test

The male children in the second grade tended to have greater BMI than the equivalent female children, and the difference tended to decrease for the older children. Considering the maximal isometric strength, the female children showed a larger increase with age than the male children (**Table 1**).

To analyze the relationships between the maximal isometric strength and the pressure parameter of tablet test, correlational analysis was performed. These data showed a positive correlation between the tablet pressure and the maximal isometric strength in the second grade male children (see **Table 4**).

**Table 4:** Correlations between tablet-computer pressure and isometric strength of arms.

Gender	Grade *(n)*	ρ	*p*
Male	II (24)	**0.48**	*0.02*
	IV (16)	0.26	*0.34*
	II+IV (40)	**0.45**	*0.003*
Female	II (25)	0.21	*0.3*
	IV (17)	0.06	*0.8*
	II+IV (42)	0.28	*0.07*

*Significant values (p < 0.05) highlighted in bold. p values reported in italic.*

### Assessment of Handwriting and the Figure-Tracing Tablet-Computer Test

#### The Figure-Tracing Tablet-Computer Test and the Sentence-Copying Test

The correlations between the four indices provided by the tablet test and the two indices provided by the DGM-P (i.e., metric variation, velocity) were examined for the male and female children (see **Table 5**). As the number of subjects was low, we can consider the reference criterion for significance as the magnitude of the correlation, rather than the *p*-value. By taking the threshold of *r* = 0.30 as this criterion, the oscillation parameter was significantly correlated with the velocity (*r* = 0.37), and was close to significant for correlation with the metric variation (*r* = 0.29), in both cases only in the male children. The other notable correlation was between pressure and the metric variation (*r* = 0.37), here only for the female children.

**Table 5:** Correlations between tablet-computer parameters and DGM-P spatial-metric variation and velocity (*z*-scores).

	Strokes		Oscillations		Pressure		Speed	
	ρ	*p*	ρ	*p*	ρ	*p*	ρ	*p*
**Spatial-metric variation**								
Male	-0.18	*0.3*	0.29	*0.10*	-0.16	*0.38*	0.20	*0.2*
Female	-0.10	*0.5*	0.04	*0.79*	**0.37**	*0.02*	-0.04	*0.8*
**Velocity**								
Male	-0.08	*0.66*	**0.37**	*0.03*	-0.26	*0.12*	0.11	*0.54*
Female	-0.06	*0.68*	0.09	*0.56*	-0.19	*0.22*	0.05	*0.73*

*Significant values (p < 0.05) highlighted in bold. p values reported in italic.*

A second analysis was carried out between oscillations (tablet test) and the two global indices of the DGM-P (i.e., legibility of letters, spatial metric variation) and two further specific indices of the DGM-P (i.e., number of fluctuant letters, average amplitude of fluctuation) that we expected to be connected to oscillations in the tablet test. The analysis was carried out separately for the male and female children, and the indices of the DGM-P (i.e., legibility, metric variation) were taken as raw scores, and not as Z scores (see **Table 6**). The data here confirmed the association between number of fluctuant letters and oscillations, although only in the male children. The correlation between spatial metric variation and oscillations, and the correlation between amplitude of fluctuation and oscillations, were also significant only in the male children.

**Table 6:** Correlations between tablet-computer oscillations and ‘amplitude of fluctuation’ of ascendant-descendent letters (index also grouped in Spatial-metric variation) and number of ‘fluctuant letters’ (index grouped in Legibility of letters) reported as raft values (not *z*-scores).

	Oscillations	
	ρ	*p*
**Legibility of letters**		
Male	0.23	*0.19*
Female	0.22	*0.17*
**Fluctuant letters**		
Male	**0.34**	*0.04*
Female	0.006	*0.9*
**Spatial-metric variation**		
Male	**0.41**	*0.02*
Female	0.08	*0.6*
**Amplitude of fluctuation**		
Male	**0.54**	*0.001*
Female	0.10	*0.5*

*Significant values (p < 0.05) highlighted in bold. p values reported in italic.*

#### The Figure-Tracing Tablet-Computer Test and the Praxis Test

None of the indices of the tablet test were correlated with the praxis of writing subtest of the BVSCO-2 test.

### Assessment of the Figure-Tracing Tablet-Computer Test and the Visual–Motor, Visual–Perceptual, Memory, and Planning Processes

#### The Figure-Tracing Tablet-Computer Test and the Visual–Motor Integration Test

By using the above criterion (i.e., *r* = 0.30) for the significance, there were two notable correlations here: between the oscillation parameter of the tablet test and the visual–motor integration score in the male children (*r* = -0.45), and between the speed parameters of the tablet test and the visual–motor integration, again in the male children (*r* = -0.33) (see **Table 7**).

**Table 7:** Correlations between tablet-computer parameters and cognitive test.

	Strokes		Oscillations		Pressure		Speed	
	rho	*p*	rho	*p*	rho	*p*	rho	*p*
**Visual–motor integration**								
Male	0.19	*0.25*	**-0.45**	*0.004*	0.05	*0.76*	**-0.33**	*0.04*
Female	-0.09	*0.6*	0.08	*0.6*	-0.27	*0.09*	-0.03	*0.85*
**Rover task**								
Male	**0.35**	*0.02*	**-0.36**	*0.02*	-0.25	*0.1*	**-0.43**	*0.006*
Female	**0.30**	*0.05*	**-0.35**	*0.02*	-0.28	*0.07*	**-0.43**	*0.005*
**Triangle task**								
Male	-0.16	*0.31*	0.07	*0.67*	-0.17	*0.3*	-0.02	*0.63*
Female	-0.15	*0.3*	-0.01	*0.97*	0.05	*0.7*	-0.01	*0.94*

*Significant values (p < 0.05) highlighted in bold. p values reported in italic*.

#### The Figure-Tracing Tablet-Computer Test and the Kaufman Assessment Battery for Children for Cognitive Functions

The four tablet-test parameters were correlated with two tasks in the KABC-II: the Rover task, and the Triangle task. These data showed that the oscillation and speed parameters of the tablet test were negatively correlated with the Rover task, while the stroke parameter was positively correlated, both in the male and female children (see **Table 7**).

#### Prediction of the Oscillation Parameter in the Male and Female Children

We applied linear regression to investigate the prediction of the oscillation parameter by the visual–motor integration and the Rover task (**Table 8**). These data showed that the visual–motor integration and the Rover task were both predictors of the oscillations in the male children, while only the Rover task predicted the oscillations in the female children. **Table 8** shows the beta levels of the visual–motor integration and the Rover task as separate variables, due to their strong interconnection.

**Table 8:** Beta parameters (standardized) of the linear regression using the tablet-computer oscillation parameter as the dependent variable, and visual–motor integration and Rover as separate independent variables.

	VMI		Rover	
	β	*p*	β	*p*
Male	-0.37	*0.02*	-0.35	*0.02*
Female	0.07	*0.66*	-0.34	*0.02*

*Male n = 39; female n = 42.*

## Discussion

Acquisition of figure-tracing skills is critical for the development of handwriting and drawing skills during school age. We analyzed figure-tracing skills using a tablet-computer test with 7–9-year-old children. The tablet test uses a digital computerized tool, and it entails the tracing of lines of simple geometrical figures, according to the trajectories illustrated on the tablet. The tablet test allows the measurement of grapho-motor parameters of figure tracing in real time. We analyzed both the psychological and physiological processes that studies have suggested to be related to graphics activities, and compared younger/older and male/female children based on specific hypotheses.

As described in the literature, the writing abilities of children develop during primary school and are consolidated at the end of this period, when automation of the hand movements is accomplished. In the last years of primary school, children develop their writing skills in a personal manner, and it can be difficult to follow their progression. Studies have also shown differences between male and female children in the development of visual cognition and in the relationships between visual cognition and other processes. Visual processes are associated with reading in male children, but not in female children (Hystegge et al., 2012), and gender differences have also been observed for drawing (Lange-Küttner and Ebersbach, 2012) and handwriting (Tabatabaey-Mashadi et al., 2015). Therefore, we analyzed the data in the present study by considering the age and the gender as relevant factors in the development of handwriting skills.

With reference to our first hypothesis, the data essentially confirmed our predictions. First, the four main parameters of number of strokes, oscillations, pressure, and speed provided by the tablet tests were partially correlated between themselves. Indeed, the speed parameter was positively correlated with oscillations and negatively correlated with number of strokes. Here, there was a trade-off between speed and oscillations: increased speed resulted in decreased quality of tracing, in terms of more oscillations. At the same time, increased speed resulted in reduction of the number of strokes. Secondly here, for the roles of age and sex for the oscillation parameter of the tablet test, there was a significant main effect of age and a significant effect of interactions, while the main effect of sex was not significant. In other words, there was a reduction in oscillations with age, but only in the male children. This implies that during this period, in male children there are changes in some processes related to movement control that allow them to reduce the oscillations.

The second hypothesis was that greater pressure on the tablet by the children was associated with higher isometric force in the arm. This was confirmed only in the male children of the second grade in the primary schools. The younger male children who showed more strength also exerted more pressure on the tablet. This can be linked to the skeletal muscle mass that will be more developed in the younger male children with respect to the female children, as also suggested by the BMIs of the second-grade male children. This difference might disappear later, as for the female children the maximal isometric strength grows faster than for the male children (De Ste Croix, 2007).

The third hypothesis was about the relationships between the figure-tracing tablet test and the paper and pencil handwriting tests. We expected to find relationships for the DGM-P writing test (e.g., copying sentences) and weaker relationships with the writing fluency task (i.e., the praxis subtest of BVSCO). For the DGM-P test, three categories of measures were considered: speed, spatial-metric variation, and legibility of letters (measured on the basis of formal aspects), and two analyses were carried out. The first analysis concerned the correlations between two indices of the sentence copying test (i.e., metric variation, speed) and the four parameters of the tablet test. The data showed that oscillations (tablet test) are correlated with velocity (DGM-P), and tend to correlate with metric variations, but only in the male children. This thus suggests that male children make use of visual–spatial integration skills as a predominant feature for solving these tasks, with respect to the female children. In addition, there was a correlation between pressure (tablet) and metric variation (DGM-P), although only in the female children. The pressure used during writing can represent an index of the automation of the writing, although if this becomes excessive, it represents an index of stiffness. We suggest here that difficulties in maintaining spatial relationships with respect to the line of writing and to the size of the letters is linked to an increase in the pressure that can be explained by immature motor control during the writing task. However, this process specifically involves the female children. The second analysis involved two specific indices of the DGM-P, as the number of fluctuant letters and the average amplitude of the fluctuations, and the oscillations for the tablet test. The results confirmed a significant correlation, but only in the male children. These results confirm the presence of common processes in the two tasks (i.e., DGM-P, tablet test). For the handwriting fluency (praxis test), we expected weak correlations with the tablet parameters, although no correlations were seen. The reason for this discrepancy might be that the praxis test involves the recognizing of the letters, not their formal precision, as for the DGM-P.

The fourth hypothesis was about the relationships between the figure-tracing tablet test and visual/ cognitive processes. We expected relationships between more general cognitive processes and the figure-tracing tablet test (Oscillation index) for both the male and female children, while we also expected visual processes to be associated with tablet parameters only in the male children. For the visual–motor integration, the data confirmed our hypothesis. The oscillations on the tablet were negatively correlated with visual–motor integration performance, but only for the male children. This means that the male children with higher visual–motor integration skills are better able to precisely control the movement of the arm during the production of spatial trajectories. Another result involved the negative correlation between speed and visual–motor integration in the male children, but not in the female children. This means that in the tablet tests, when the children were given the ‘best condition’ instruction, for the male children, their ability to reduce their speed was associated with better visual–motor integration skills. For the KABC-II, the four parameters of the tablet test were correlated with two tasks: Rover and Triangles. The Rover task involves the planning of a spatial route, while the Triangles task is a classical visual-constructive task (i.e., putting together the pieces to form a specific pattern). These results show that three of the tablet parameters were correlated with the Rover task: stroke (positively), oscillations (negatively), speed (negatively); however, none of the tablet parameters were correlated with the Triangle task. This means that the general visual cognitive processes (i.e., Rover task) were correlated with tablet performance for both sexes, while the visual-constructive processes were not involved in the tablet test.

Due to the correlations that were revealed with respect to the two different processes of visual–motor integration and visual cognition (or spatial planning; Rover), their respective influences on the quality (oscillations) of the figure tracing in the male and female children were analyzed. Regression analysis showed different results across the two sexes. In the female children, visual cognition explained a significant part of the variance in the oscillations on the tablet, while the visual–motor integration was not involved. On the other hand, for the male children, both visual cognition and visual–motor integration explained a significant part of the variance in the oscillations on the tablet, but they were not independent. In the male children, the two factors largely explain the same variance in the tablet oscillations. Therefore, it appears that the male and female children use different processes to improve the quality of their performance with the tablet. While the male children make use of visual–spatial and visual–motor integration skills to reduce the oscillations, the female children make use of more general visual cognition processes.

## Conclusion

The data from the present study extend previous studies concerned with the use of digitizer-based measures to assess handwriting skills development. Here, the use of a simple figure-tracing tablet-computer test was suggested to investigate some specific aspects of this ability. First, the tablet test saves time in the acquisition of the data with respect to paper and pencil tests, and it provides interesting correlations. Moreover, it is a sensitive tool in the analysis of the gradual development of handwriting skills by children, and it is associated with variables that are relevant for these skills. Moreover, in agreement with Smits-Engelsman and van Galen (1997), who reported that poor writers lack control of spatial accuracy, we believe that complex stimuli are not necessary to study these basic processes, as simple stimuli are sufficient. However, the picture that emerged from the present study revealed things to be relatively complex because the differences in the processes involved in handwriting skills development are related to age and gender. Therefore, there still remains much to do in future studies.

For now, although the data from the tablet test are still relatively basic, they were partially correlated with the data from the paper and pencil writing tests. Based on this association, the tablet oscillation parameter appears to represent the most useful index for assessing of the processes under scrutiny here.

## Author Contributions

EG has contributed to the implementation of the software and data acquisition on the tablet, analyzed the data acquired with the tablet. SDS, TA, GF-I, TP contributed to the design of the entire study. SDS, TA, GF-I, and TP collaborated to write the documents for the approval by local Ethics Committee. SDS and PC contributed to subject recruitment. PC collected and organized the paper–pencil tests. SDS and TP analyzed the overall data and drafted the manuscript. EG, SDS, TA, PC, GF-I, and TP reviewed and approved the manuscript.

## Conflict of Interest Statement

The authors declare that the research was conducted in the absence of any commercial or financial relationships that could be construed as a potential conflict of interest.
